# Mitral valve dynamics in structural and fluid–structure interaction models

**DOI:** 10.1016/j.medengphy.2010.07.008

**Published:** 2010-11

**Authors:** K.D. Lau, V. Diaz, P. Scambler, G. Burriesci

**Affiliations:** a Centre for Mathematics and Physics in the Life Sciences and Experimental Biology (CoMPLEX), UCL, United Kingdom; b UCL Institute of Child Health, United Kingdom; c Department of Mechanical Engineering, UCL, United Kingdom

**Keywords:** Biomechanics, Finite element, Fluid–structure interaction, Heart valves, Mitral valve

## Abstract

Modelling and simulation of heart valves is a challenging biomechanical problem due to anatomical variability, pulsatile physiological pressure loads and 3D anisotropic material behaviour. Current valvular models based on the finite element method can be divided into: those that do model the interaction between the blood and the valve (fluid–structure interaction or ‘wet’ models) and those that do not (structural models or ‘dry’ models).

Here an anatomically sized model of the mitral valve has been used to compare the difference between structural and fluid–structure interaction techniques in two separately simulated scenarios: valve closure and a cardiac cycle. Using fluid–structure interaction, the valve has been modelled separately in a straight tubular volume and in a U-shaped ventricular volume, in order to analyse the difference in the coupled fluid and structural dynamics between the two geometries.

The results of the structural and fluid–structure interaction models have shown that the stress distribution in the closure simulation is similar in all the models, but the magnitude and closed configuration differ. In the cardiac cycle simulation significant differences in the valvular dynamics were found between the structural and fluid–structure interaction models due to difference in applied pressure loads. Comparison of the fluid domains of the fluid–structure interaction models have shown that the ventricular geometry generates slower fluid velocity with increased vorticity compared to the tubular geometry.

In conclusion, structural heart valve models are suitable for simulation of static configurations (opened or closed valves), but in order to simulate full dynamic behaviour fluid–structure interaction models are required.

## 1 Introduction

Located between the left atrium and left ventricle of the heart, the mitral valve ensures unidirectional flow of blood from atrium to ventricle and is characterised by an asymmetric bicuspid leaflet structure and the chordae tendineae: chord-like structures which tether the valve directly to the heart wall, preventing inversion of the valve. The mitral valve leaflets form the largest surface area of the four heart valves and are subjected to the highest pressure load during systole, a factor which contributes to the degeneration and dysfunction of a diseased valve. Correct function of the mitral valve requires the synergy of four valvular components: (1) the annulus, (2) the leaflets, (3) the chordae tendineae and (4) the papillary muscles. Valvular dysfunction arises from functional changes in the ultrastructure in any of the components, which can be caused by a range of factors such as infection, variability in the circulatory system or old age [7]. The mitral valve controls the unidirectional flow of oxygenated blood into the left ventricle from the left atrium and changes in valve function have a global effect on the cardiovascular system. Depending on the severity of the loss of function, a heart valve replacement or corrective valvular repair (such as annuloplasty or the edge-to-edge technique) is required to restore functionality of the valve.

The biomechanics of the mitral valve is a complex problem due to the large deformations of the structure, 3D anisotropic non-linear elastic behaviour of the valvular tissue and pulsatile haemodynamic loads during the cardiac cycle. Imaging techniques such as magnetic resonance imaging and echocardiography [3] allow the visualisation of the heart valves, linking anatomy to valvular function, but do not yield any information on the loads applied to the valve, nor the coupled fluid and structural dynamics of the valve and the blood; therefore, modelling is the most suitable technique method of analysing valve function. It is hoped that with the development and simulation of anatomical models under physiological conditions it will be possible to gain a qualitative insight into valvular function and the suitability of different healthcare options for diseased valves.

Models of the mitral valve can be broadly divided into two groups: (1) structural models which only contain the structural domain of the valve and (2) fluid–structure interaction (FSI) models which include both the fluid domain of the blood and the structural domain of the valve. In a ‘dry’, structural model the transvalvular pressure load is applied directly to the valve structure as a boundary condition and has been modelled as uniformly distributed over the surface in all current models. In a ‘wet’, FSI model the two domains are coupled together and corresponding dynamics are solved simultaneously (changes in the structural domain affect the fluid domain and vice versa); the pressure load applied to the valve structure is defined from the coupling applied to the two domains, not the boundary condition applied to the model, and is non-uniform.

Numerical studies of the mitral valve have been performed by a number of authors, with each using modelling to examine the role of different valvular aspects; for example the role of the chordae tendineae [13] or the effect of annular dilation and correction [16] have both been previously studied using structural models. Currently the only FSI model of a native mitral valve available in the literature has been developed by Kunzelman et al. [10], this work was characterised by positioning the valve in a tubular fluid volume in which valve closure was simulated. However as only closure was performed no details regarding the dynamics of opening/closure were presented, with comparisons between FSI and structural models made by only comparing the stress-state of the valve in a closed configuration.

Hence the objective of this work is to verify how the predicted valve behaviour is affected by the difference in modelling techniques currently used, with particular emphasis on the difference in valvular dynamics between structural and FSI techniques and the influence of the fluid volume on the valve. Using a model of the mitral valve, simulations of both valve closure and the entire cardiac cycle have been performed in “dry” (structural only) and “wet” (FSI) scenarios using a commercially available finite element code. As previous FSI studies of the mitral valve have been performed solely in tubular geometries, in this work the effect of the fluid volume on valve dynamics and vice versa is quantified with FSI simulations in both tubular and ventricular-shaped fluid volumes. This has particular implications for hydrodynamic testing of artificial valves, as these are performed in straight fluid chambers as required by current international regulations (ISO 5840:2005 [8] and FDA Replacement Heart Valve Guidance).

In order to simplify the complexity of the problem, certain aspects of the valve model have been simplified: the valve leaflets and chordae tendineae have been modelled as linear elastic materials, neglecting their non-linear constitutive behaviour and anisotropy, and the surrounding tissues (including the ventricular muscle) have been assumed to be rigid.

## 2 Methods

The structural and fluid dynamics of the mitral valve and the surrounding blood were simulated using the explicit finite element code LS-DYNA [5]. The geometry and finite element mesh of all the models described in this work were generated using ANSYS (Release 11.0) and subsequently converted to a LS-DYNA compatible format using a custom written script. All the numerical simulations were performed on a Intel Xeon 2.66 GHz workstation operating with LS-DYNA 971 Release 3.2.1. Post-processing of the data was performed with LS-PREPOST and Ensight.

### 2.1 Mitral valve model

**Fig. 1:**
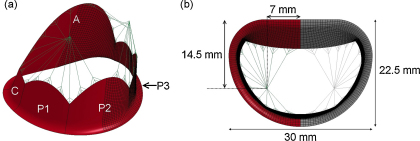
(a) Profile of the mitral valve model, with and without the finite element shell mesh; leaflet labels: A, anterior leaflet; C, commissural leaflet; P1/P3 and P2, side and central cusps of the posterior leaflet. (b) Model profile as viewed from the left atrium with the anteroposterior (22.5 mm) and commissural (30 mm) diameters labelled; the reflected component of the geometry is represented in grey.

The different components of the mitral valve were modelled using anatomical measurements and descriptions from the literature [6,14]. The initial geometry of the valve model was defined with the leaflets in an open configuration and in order to reduce the computational time required, only half the model was simulated; the plane of symmetry was defined as the anteroposterior axis of the valve, as shown in Fig. 1. A summary of the model parameters is listed in Table 1.

The annulus of the model has been defined as a planar D-shaped orifice, which is fixed spatially. The annular proportion and length of the different cusps have been defined from anatomical measurements of excised human valves [14]. Studies on porcine mitral valves have shown that the annular length of an intact valve is shorter than that of an excised valve [9], for this reason the annular length of each cusp segment has been adjusted from the reported excised value and scaled to the corresponding annular segment of the model. The total annular length of the model is 85.7 mm and the annular lengths of the different leaflet sections are listed in Table 1. The leaflets of the valve model extend outwards from the annulus towards the free margin of the leaflets, which has been defined using splines connecting the midpoint of each cusps (defined as the leaflet height) to an interface point, defined as 7.5 mm above the annular plane, between each cusp.

In this model only the primary chordae tendineae, which are attached to the free margin of the leaflet, have been modelled. The number and location of these chordae have been based on anatomical descriptions [11,14]. These chordae have been modelled as attached to a static node which represents the papillary muscle tip, positioned in a plane 20.0–22.0 mm above the annular plane (Fig. 1). The location of papillary tip of each chordae tendineae was adjusted to ensure coaptation of the leaflet occurs with the application of physiological pressure.

#### 2.1.1 Mitral valve finite element model

The geometry of the valve model was discretised into a finite element mesh of shell elements for the leaflets and beam elements for chordae tendineae. The finite element shells and beams used in this model were Belytschko--Tsay shell elements and Hughes--Liu beam elements respectively. The beam elements have been modelled as cables, which collapse under compression as they generate no force. The physical properties of the shell and beam elements are listed in Table 2 and the density of all elements was set to 1000 kg m^−3^.

The independence of the finite element solution to the size of the elements in the valvular model was determined independently of the fluid model. A simulated pressure ramp of 120 mmHg (∼16 kPa) over 300 ms was applied and sustained on the structure of the model until the solution converged. These simulations were run using a global nodal damping factor in LS-DYNA that was set to 0.9965, a value which was based on previous experience [1]. The simulations were considered converged when the current global kinetic energy *E*_ke_ was reduced to a value smaller than the maximum of the global kinetic energy $E_{\text{ke}}^{\text{max}}$ multiplied by the convergence tolerance factor, here set to 1 × 10^−6^. The solution was considered independent when no observable difference in the stress pattern was noted and the difference in the value of the peak principal stress was less than 1% between models. The final model consisted of 2688 shell elements and 333 beam elements (Fig. 1).

### 2.2 Fluid volumes: tubular and ventricular models

**Fig. 2:**
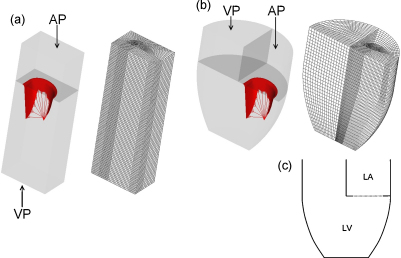
(a) Tubular fluid volume and mesh and (b) ventricular fluid volume and mesh; figure labels AP and VP denote the atrial and ventricular pressure surfaces respectively. (c) Schematic outline of the ventricular fluid volume with the solid boundaries (solid lines) and annular orifice (dotted line) labelled.

The valve model has been inserted and simulated within: (1) a tube-shaped fluid volume and (2) a ventricular-shaped fluid volume. These fluid volumes are static and rigid, with a fixed volume. The geometry of these fluid volumes represent the left ventricle of the heart only and have been sized on the volumetric measurements of the left ventricle. As the FSI simulations include aspects of systole and diastole, an intermediate volume of 35 cm^3^ for the atrial section and 85 cm^3^ for the ventricular section was chosen. The fluid volume geometries and the surfaces used to apply the transvalvular pressures are shown in Fig. 2.

#### 2.2.1 Fluid volumes: finite element models

The geometry of the two fluid volumes was discretised into solid LS-DYNA ALE fluid elements, with a mesh designed such that the valvular orifice matched the shape of the valve annulus (Fig. 2). The fluid was modelled as a Newtonian fluid and was defined with blood-like properties (see Table 2) and a density of 1000 kg m^−3^. As the fluid model of LS-DYNA is compressible, the bulk modulus must be defined. In an explicit finite element formulation this factor controls the size of the maximum timestep of the simulation; thus, to reduce the required simulation time the value of the the bulk modulus was reduced to 1% of its physical value, following [10]. This was determined not to affect the solution of the simulation by comparison to the analytical solution to infinite channel flow.

As with the structural mesh, the element size of the fluid mesh was selected such that the independence of the solution to the mesh was obtained. This was performed by applying a velocity boundary condition to the inlet of each model equal to 1 m s^−1^. The solution was considered converged when the absolute difference in the velocity between subsequent timesteps was of the order of 10^−6^. Once stabilised, the velocity across the valve plane was compared until the difference between subsequent models was reduced to less than 5% in the region of interest. The final models of the tubular and ventricular volumes consisted of 81,920 and 43,008 solid brick elements respectively, as shown in Fig. 2. The ventricular pressure plane (VP) was defined at the plane of the outlet of the fluid volume in both FSI models.

### 2.3 Transvalvular pressure load

**Fig. 3:**
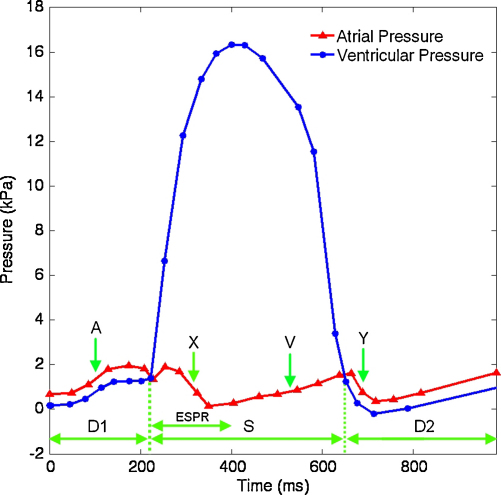
Transvalvular pressure load, values after [12]. The ventricular closing pressure curve section is defined in grey. D1, 1st diastolic phase, valve open; ESPR, early systolic pressure ramp, valve closing; S, systolic phase, valve closed; D2, 2nd diastolic phase, valve open; A, atrial systole (A wave); X, atrial relaxation (X wave); V, atrial filling (V wave); Y, atrial emptying (Y descent).

Using the structural and FSI models, the structural and fluid dynamics have been simulated using two different pressure loads: (1) early systolic pressure ramp (ESPR) simulating valve closing and (2) a full cardiac cycle simulating both valve opening and closing (see Fig. 3) [12]. For the early systolic ramp, the transvalvular pressure was measured from the point of the left atrial/left ventricle pressure cross-over until the maximum of ventricular pressure. The maximum ventricular pressure was sustained for a further 22 ms, in order to ensure the valve was in a static configuration, taking the total simulation time to 200 ms.

#### 2.3.1 Structural simulation

The transvalvular pressures described in Fig. 3 were applied directly to the structural model as a uniform pressure load distributed over the corresponding atrial and ventricular sides of the leaflet structure. As before, the nodal velocities of the valve structure was scaled using a global damping factor (see Section 2.1.1). In order to model the valve as a single surface structure, a contact penalty condition was applied to all the shell elements of the model.

#### 2.3.2 Fluid–structure simulation

In the FSI simulations, the transvalvular pressure was applied to the boundaries of the fluid models (see Fig. 2) and the resulting fluid flow has been coupled to the structure of the valve by a penalty coupling method: if the fluid contacts and penetrates the structure of the valve, a resistive force linearly proportional to the distance penetrated is applied to both the fluid and the structure, thus coupling them together.

Under the application of the ventricular pressure ramp (Fig. 3) the fluid–structure coupling has only been applied to the ventricular side of the valve, as fluid only approaches from this side. In the full cardiac cycle simulation this coupling was applied to both atrial and ventricular sides of the valve, as fluid flow approaches the valve from both atrial and ventricular sides.

Fluid flow was restricted to flow through the valve by applying a no-slip condition to the nodes in the plane of the valve. The boundaries of the volume, with the exception of the inflow and outflow sections (AP and VP in Fig. 2), were also defined as no-slip.

## 3 Results

### 3.1 Valve closure

**Fig. 4:**
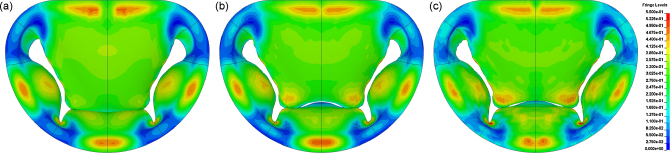
Maximum principal stresses in the valve at *t* = 200 ms in units of MPa in: (a) the structural model, (b) the tubular model and (c) the ventricular model. Stress range from 0 to 550 kPa.

**Fig. 5:**
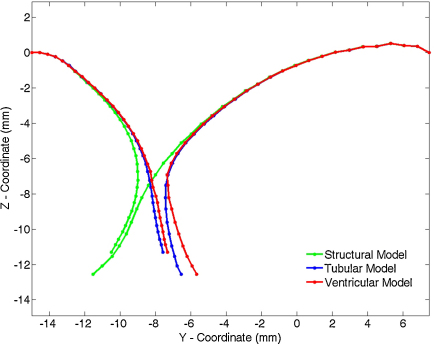
Configuration of the valve in the anteroposterior section at *t* = 200 ms closure in the structural, tubular and ventricular models.

Under the application of the ventricular pressure curve valvular closure was simulated. The valve leaflets coapt together and remain closed until the end of the simulation. Here the structural and FSI models have been analysed and compared at closure, when the structure is in a static, closed configuration. The maximum values of the principal stresses at *t* = 200 ms and their locations in the three different models are listed in Table 3. The corresponding stress distribution in the valve, as viewed from the ventricular side, is presented in Fig. 4. The configuration of the valve, represented by the nodes in the anteroposterior section, at *t* = 200 ms is displayed in Fig. 5.

### 3.2 Cardiac cycle

**Fig. 6:**
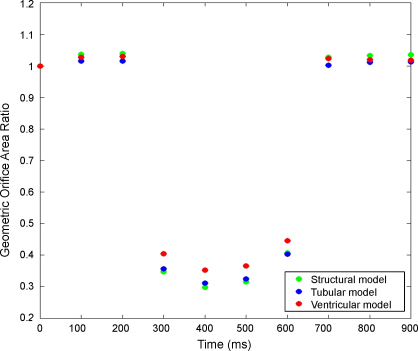
Variation in the geometric orifice area (GOA) during the cardiac cycle for the structural and FSI models. Ratio measured against initial orifice area at *t* = 0 ms.

Under the application of the cardiac cycle pressure curve valvular opening, closing and reopening was simulated. The results of the two FSI simulations have been compared against each other and also with the structural simulation subject to the same pressure load. Table 4 lists the maximum resultant nodal velocity in the anteroposterior section of the valve during opening and closing, accompanied with the time of occurrence. Fig. 6 illustrates the corresponding valve configuration and nodal velocity during the closing phase at the time of maximal nodal velocity in each model.

**Fig. 7:**
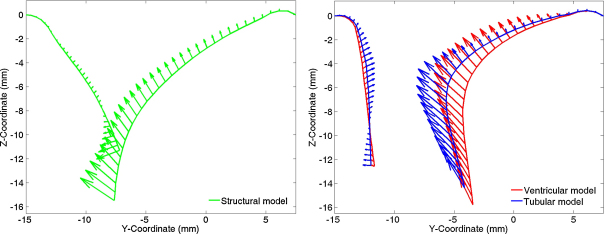
Valve configuration, through the anteroposterior section, at the time of maximum nodal velocity (see Table 4) in: (a) the structural model and (b) the tubular and ventricular models. Here the nodal positions have be drawn with the corresponding velocity vectors.

Fig. 7 illustrates the change in geometric orifice area (GOA) of the different models throughout the simulated cardiac cycle. The GOA was determined by measuring the area enclosed by the valve leaflets, relative to a plane parallel to the annulus at the height of the commissural/anterior leaflet cleft, at 100 ms intervals during the cycle.

**Fig. 8:**
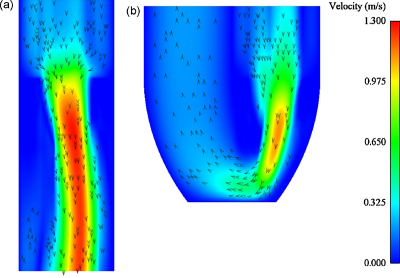
Tubular and ventricular fluid velocity fields, in the symmetry plane, during diastolic filling phase (D1) at *t* = 200ms. Velocity range from 0 to 1.3 m s^−1^.

In the fluid domain, the change of sign in the transvalvular pressure gradient causes the flow to change direction between the D1, S and D2 phases of the pressure curve (see Fig. 3). In the D1 phase of the cardiac cycle, the valve is open and flow passes from the atrial chamber to the ventricular chamber; the flow field during this phase (*t* = 200 ms) in the tubular and ventricular models, as viewed through the symmetry plane of the fluid volume, is shown in Fig. 8.

**Fig. 9:**
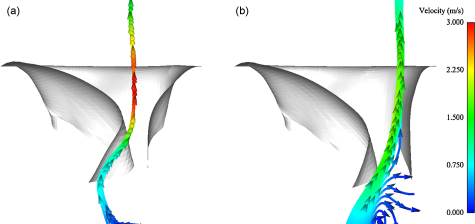
Fluid particle trace during valve closure at *t* = 257 ms in: (a) the tubular model and (b) the ventricular model. Velocity range from 0 to 3.0 m s^−1^.

Following the D1 phase is the S phase, during which the flow direction reverses causing the valve to close. The closure of the valve generates a fluid jet, or dynamic regurgitation, forcing fluid back into the atrial section. The timing and maximum magnitude of the nodal *z*-velocity in the valvular plane in the tubular and ventricular models is listed in Table 5. A particle trace following the velocity field of the fluid in the tubular and ventricular models at *t* = 257 ms is presented in Fig. 9.

**Fig. 10:**
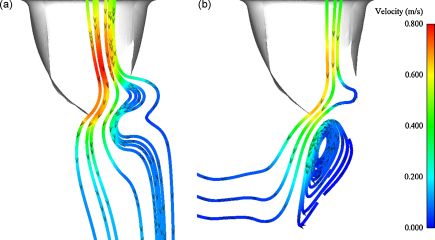
Fluid particle trace during valve opening at *t* = 685ms in: (a) the tubular model and (b) the ventricular model. Velocity range from 0 to 0.8 m s^−1^.

After closure of the valve in the S phase, the valve re-opens during the D2 phase. As flow enters the ventricular chamber of the fluid volumes, fluid mixing generates vorticity in the flow. A particle trace following the velocity field of the fluid in the tubular and ventricular models at *t* = 685 ms is presented in Fig. 10. The time points of both Figs. 9 and 10 were selected to coincide with the maximum of transvalvular velocity and when the behaviour of the fluid was most evident. The corresponding maximum vorticity at bith these timepoints (*t* = 257 ms and *t* = 685 ms) in both tubular and ventricular fluid geometries is listed in Table 6.

## 4 Discussion

### 4.1 Valve closure

The configuration of the valve at closure in the different models is compared in Fig. 5. In all of the models, the leaflets have coapted and the valve has closed. The gap in the FSI models is a numerical artifact, which has also been noted by Kunzelman et al. [10]. The nodal positions at the base of the valve compare well in all the models, but significant differences arise towards the leaflet tips between the structural and FSI models; in both the FSI models the tips of the posterior leaflets are positioned further towards the anterior side than the structural model. This difference arises from the simplification of uniform pressure applied to the surface of the valve in the structural simulation. At the base of the valve, this assumption holds well as the pressures on each side of the valve corresponds to the pressure in the atrial and ventricular chambers. Towards the tips of the leaflets, close to the coapting surfaces, the tranvalvular pressure is no longer the same as the pressure over the rest of the body and the assumption of uniform pressure no longer holds. The resulting non-uniform pressure distribution over the valve results in the difference in closure exhibited in Fig. 5.

The corresponding distribution of local (elemental) maximum of the principal stresses are compared in Fig. 4 and their global maxima at closure are listed in Table 3. Both FSI models presented higher maximum values of stress than the structural model, with the ventricular model with the largest value. In all models the peak stress were located at the fold of the posterior leaflet (P2/P3). From Fig. 4 it can be seen that the distribution of stress is similar, with stress concentrations located at the base of the cusps and at the folds of the leaflet.

For closure events only, structural models simulate a similar stress distribution to FSI models, although with a lower magnitude of stress. However structural models do have a clear advantage over FSI in terms of the required simulation time, as shown in Table 7.

### 4.2 Cardiac cycle

The difference in the nodal velocity of the valve structure, in the anteroposterior section, during opening and closing has been compared in Table 4. During closure of the valve in the S phase, the maximum nodal velocity in the structural model is comparable to the nodal velocities in the FSI simulations. As the valve reopens in the D2 phase, the maximum nodal velocity in the structural model is significantly lower than the FSI model.

The nodal coordinates and corresponding velocities of the nodes in the anteroposterior section of the valve during valve opening (S phase) of the different models are presented in Fig. 6. In the structural simulation the maximum nodal velocity occurs at the tip of the anterior leaflet when the anterior leaflet is close to impacting the posterior leaflet; whereas in the FSI simulations the maximum nodal velocities occur close to the centre of the anterior leaflet whilst the leaflets are still separated by a distance of approximately 5 mm.

From comparing the coaptation of the leaflets, subtle differences were found in the two FSI simulations. In the tubular model both leaflets were displaced to the centre of the orifice during closure, which resulted in the posterior leaflet making contact with the centre of the anterior leaflet. With the further application of pressure the point of coaptation then moved towards the anterior leaflet tip. In the ventricular model, the posterior leaflet sustains a smaller displacement and the anterior leaflet contacts the posterior leaflet close to the tip of the anterior leaflet and with the application of further pressure no further displacement occurs.

Variation in the GOA in both FSI models during the D1 and D2 phases were found to be marginally smaller than the structural model, with the ventricular model larger than the tubular model (see Fig. 7). During the S phase the structural model presented a smaller GOA than both FSI models, with all models following the same trend. However the complete closure of the valve in the FSI models is hampered by the fluid element gap, as previously highlighted in Fig. 5.

In Figs. 8–10 the differences in fluid dynamics between the FSI models have been compared. In the D1 phase, the valve begins in an open position and flow passes through the valvular orifice into the ventricular chamber (Fig. 8). In the tubular model the flow is directed to the posterior side by the anterior leaflet, but in the ventricular model the flow bends round the bottom of the ventricular volume towards the left ventricular outtract. Although the maximum velocity of the core of the fluid is similar between the FSI models, the extent of the jet is significantly smaller in ventricular model as the fluid more restricted than in the tubular model.

Following this first filling phase is the S phase during which the direction of fluid flow reverses and closes the valve. The motion of the valve during closure generates a dynamic regurgitant fluid jet in both FSI models. From measuring the velocity of the fluid nodes lying in the valvular plane of the fluid volumes, the maximum velocity was found to occur at approximately the same time but the magnitude in the tubular model was found to be approximately 50% greater than that of the ventricular model (Table 5), which correlates with the higher transvalvular flow velocities as shown in Fig. 8. Comparison of the filling velocity compares well to values described in the literature of approximately 80 cm s^−1^ [15].

After the S phase comes the D2 phase, during which the valve reopens and flow passes from the atrium to the ventricle. As the valve opens a fluid jet forms, generating vorticity in the ventricular chamber of the fluid volume. Due to the faster transvalvular flow, the maximum value of the vorticity was found to be greater in the tubular model than the ventricular model (see Table 6). However the vorticity of the flow was found to have a larger extent in the ventricular fluid volume than the tubular volume, as highlighted in the particle traces of during reopening of the valve in Fig. 10.

Analysis of the two fluid volumes used in the FSI simulations has shown that the valvular dynamics, in terms of the nodal configuration at closure and the nodal velocities during opening and closing, are comparable between the FSI models although differences were noted in the coaptation dynamics. The effect on the coupled fluid dynamics in the fluid volumes were found to generate higher velocity flow in the tubular volume during opening and closing, along with differences in the fluid vorticity during reopening.

### 4.3 Limitations

The valvular model used in this work has been useful in analysing the differences between the structural and FSI modelling techniques, but it has limitations such as the uniform thickness and the linear elastic behaviour of the valve model. Although the anterior cusp and the central posterior cusp coapt successfully, the valve does not fully close in the commissural areas. Due to the expensive computational time required, the FSI simulations presented here have only been performed for a single cycle.

The fluid volumes used to represent the left atrium and ventricle have been sized using realistic volumetric measurements, but are non-physiological in that the boundaries are modelled as rigid and static; the contractility of the LV chamber and the papillary muscles have been neglected.

However the models incorporate aspects which have not been previously examined in heart valve modelling and offer a qualitative description of the valve behaviour under different operative conditions. The same modelling techniques can also be applied to tissue [2] and polymeric [4] valves which also undergo large deformation in response to fluid loads.

## 5 Conclusion

Here the difference between ‘dry’ structural models and ‘wet’ fluid–structure interaction models have been presented in two separate simulations, one simulating closure only and the other simulating the full cardiac cycle. From the closure simulation it has been shown that the structural model is similar to the FSI models in terms of the predicted stresses but not the stress magnitude or the valvular configuration. Although there are differences between the models, the numerical complexity of a FSI simulations highlights the suitability of structural simulations for the modelling of static configurations such as closed valves.

In the cardiac cycle simulation the coupled dynamics of the structure and the fluid have been examined. It was found that the behaviour of the valve varied significantly between the structural and FSI models due to the uniform pressure distribution in the structural model; the dynamics of the valve in the two fluid volumes were found to be similar, with subtle difference in the coaptation of the valve. Significant differences in the fluid domains of the FSI models were noted, with the tubular model exhibited faster fluid velocity during both opening and closing of the valve and a reduced fluid vorticity when compared to the ventricular model.

In conclusion, structural heart valve models are more reliable for simulation of static events, but in order to accurately simulate full dynamic behaviour FSI models are required. This dynamic motion is essential in the simulation of valvular repairs, such as the edge-to-edge technique, where the valvular dynamics are affected by reduction of the effective valvular orifice and restricted motion of the leaflets themselves. Also, as the mitral valve regulates the flow in the heart, FSI models simulating normal and pathological states (such as valvular stenosis where valvular motion is restricted) are important, as they may allow qualification on the role valve motion has on the flow rate and the effect on the circulatory system.

## Conflict of interest

None.

## Figures and Tables

**Table 1:** Dimensional parameters of the mitral valve model.

Leaflet	Annular length (mm)	Leaflet height (mm)	Leaflet area (mm^2^)
Anterior	28.9	23.4	444.3
Commissural	7.0	8.7	46.1
Posterior (P2)	17.4	13.8	177.7
Posterior (P1/P3)	12.7	11.2	111.4

**Table 2:** Shell/beam/solid element properties. Density of all elements, *ρ* = 1000 kg m^−3^.

	Shell	Beam	Solid
Thickness (mm)	0.75	–	–
Cross-sectional area (mm^2^)	–	0.6	–
Elastic modulus (MPa)	3	40	–
Bulk modulus (MPa)	–	–	22
Dynamic viscosity (Pa s)	–	–	4.0 × 10^−3^

**Table 3:** Comparision of maximum value of principal stress and valvular location at valve closure.

Model	Maximum principal stress (kPa)	Location
Structural	566.4	P2/P3 fold
Tubular	626.3	P2/P3 fold
Ventricular	635.3	P2/P3 fold

**Table 4:** Maximum anteroposterior nodal velocity during closing (C) and opening (O) phases in the structural, tubular and ventricular models, listed with the time of occurrence.

Model	C/O	Time (ms)	Max. resultant velocity (m s^−1^)
Structural	C	246	0.922
Tubular	C	254	1.202
Ventricular	C	252	1.058

Structural	O	664	0.351
Tubular	O	684	0.748
Ventricular	O	684	0.657

**Table 5:** Time and maximum magnitude of the nodal fluid *z*-velocity in valvular plane of the tubular and ventricular models.

Model	Time (ms)	Max. *z*-velocity (m s^−1^)
Tubular	255	2.738
Ventricular	254	1.857

**Table 6:** Maximum magnitude of the vorticity during closing (C) and opening (O) phases in the tubular and ventricular models in the anteroposterior plane, listed with the time of occurrence.

Model	C/O	Time (ms)	Max. of vorticity (s^−1^)
Tubular	C	257	1.21 × 10^−3^
Ventricular	C	257	0.79 × 10^−3^

Tubular	O	685	0.31 × 10^−3^
Ventricular	O	685	0.35 × 10^−3^

**Table 7:** Structural and FSI model simulation time for the different pressure loads.

Model	Pressure load	Approx. simulation time
Structural	Closure	6 min
Tubular	Closure	18 h
Ventricular	Closure	13 h

Structural	Cardiac cycle	50 min
Tubular	Cardiac cycle	90 h
Ventricular	Cardiac cycle	65 h
